# Dog-Mediated Rabies Surveillance in Nigeria (2014–2023): Investigating Seasonality and Spatial Clustering

**DOI:** 10.3390/tropicalmed10030076

**Published:** 2025-03-12

**Authors:** Rebecca D. Williams, Mahbod Entezami, Ruth Alafiatayo, Olaniran Alabi, Daniel L. Horton, Emma Taylor, Rachel Tidman, Columba T. Vakuru, Taiwo Olasoju, Abel B. Ekiri, Joaquin M. Prada

**Affiliations:** 1 School of Veterinary Medicine, University of Surrey, Daphne Jackson Road, Guildford GU2 7AL, UKme00286@surrey.ac.uk (M.E.);; 2 Department of Veterinary and Pest Control Services, Federal Ministry of Agriculture and Rural Development, Area 11, Garki, Abuja 900103, FCT, Nigeria; 3 Science Department, World Organisation for Animal Health, 75017 Paris, France

**Keywords:** epidemiology, Nigeria, rabies, surveillance, spatial modelling

## Abstract

Rabies is an important zoonotic disease responsible for 59,000 human deaths worldwide each year. More than a third of these deaths occur in Africa. The first step in controlling rabies is establishing the burden of disease through data analysis and investigating regional risk to help prioritise resources. Here, we evaluated the surveillance data collected over the last decade in Nigeria (2014–2023). A spatio-temporal model was developed using the NIMBLE (1.2.1) package in R to assess outbreak risk. Our analysis found a high risk of canine rabies outbreaks in Plateau state and its surrounding states, as well as increased trends of outbreaks from July to September. The high number of reported canine rabies outbreaks in the North Central region could be due to cross-border transmission or improved reporting in the area. However, this could be confounded by potential reporting bias, with 8 out of 37 states (21.6%) never reporting a single outbreak in the period studied. Improving surveillance efforts will highlight states and regions in need of prioritisation for vaccinations and post-exposure prophylaxis. Using a One Health approach will likely help improve reporting, such as through integrated bite-case management, creating a more sustainable solution for the epidemiology of rabies in Nigeria in the future.

## 1. Introduction

Rabies is a globally important zoonotic disease, caused by the single-stranded negative-sense RNA rabies virus. It is transmitted by the saliva of an infected animal (RABV) by bite or licking of an open wound or mucous membranes. Canine-mediated rabies results in approximately 59,000 annual human deaths globally, 36.4% of these in Africa [1]. It is a preventable disease, yet it is currently endemic in Nigeria [2,3], with a marked rise in dog-positive cases reported at the end of the 20th century [4]. Rabies is a high public health risk causing acute fatal progressive encephalitis [5]. In a review of human deaths from dog-mediated rabies across 10 Nigerian states, there were only 78 deaths reported between 1980 and 2014. These were confirmed by clinical presentation alone, and not flaboratory techniques, due to religious and cultural concerns [6]. Another study based in the capital of Enuga State showed 149 presentations of dog bite cases to the University of Nigeria Teaching Hospital from 2004 to 2013, with six of these cases being diagnosed with rabies—all of which were fatal [7]. Both studies suggest that the true number of human deaths from rabid dog bites is likely much higher, with the number of rabies cases being grossly under-represented, potentially due to misdiagnosis or victims dying in remote communities without healthcare access, education, or awareness. These figures highlight the importance of surveillance and understanding both the epidemiology of rabies and the burden of disease.

Currently, a passive surveillance system is implemented in Nigeria for animal rabies. The central animal health laboratory, the National Veterinary Research Institute (NVRI), based in Plateau State, will promptly notify the office of the Chief Veterinary Officer of Nigeria (CVON) and the respective state Director of Veterinary Services (DVS) upon confirmation of a rabies case. This information is cascaded down to the surveillance agent and the affected community at the local government level, which can potentially inform health-seeking behaviour. Supplementary Figure S1 displays the process of animal disease reporting in Nigeria. While this is successful to a degree, not all rabid animals come into contact with humans and when they do, it is not always reported [8,9]. Furthermore, reports of animal-to-human interactions may not necessarily get reported through the local governments to the state level or national and international agencies, reducing the effectiveness of the surveillance system and underestimating the impact of the disease. Ineffective surveillance at the community level means that there is limited or no informing of exposed people in the community, resulting in either completely preventable deaths and/or inappropriate treatment/use of pre-exposure prophylaxis (PrEP) or post-exposure prophylaxis (PEP), if available and accessible. Moreover, considering the limited resources, policymakers may not prioritise rabies or make those resources available when few cases are being reported. In Nigeria, detailed rabies outbreak records are generally available at local levels and at the state level in the office of the DVS, with individual dates and locations recorded. Data are then aggregated when reporting to the central government, in this case, the Federal Ministry of Agriculture and Rural Development (FMARD). Nevertheless, the dataset is spatially explicit (at the state level), with a monthly temporal resolution, so it can be used to assess spatiotemporal relationships.

We first carry out a descriptive statistical analysis on the available dataset and assess potential spatial or temporal associations in the frequency of canine rabies outbreaks across the different Nigerian states. Through the use of a spatio-temporal model, we analyse the spatial and temporal trends of outbreaks across the states of Nigeria between 2014 and 2023. We then discuss the needs of the rabies surveillance programme, and what changes could be implemented to help with the elimination of canine rabies. By identifying limitations and suggesting improvements, the authorities will be better equipped to develop appropriate plans, including enhanced surveillance, dog vaccination programmes, and appropriate use of PEP, building towards the ‘Zero by 30’ goal of zero human deaths as a result of dog-mediated rabies by 2030 [10] and the ultimate elimination of canine rabies in Nigeria.

## 2. Materials and Methods

### 2.1. Study Population

The target population was the at-risk *Canis familiaris* population in Nigeria based on the susceptible population. The definitions used here for the susceptible population and confirmed cases are the definitions used by the FMARD. The susceptible population is taken as the number of dogs in the local environment, estimated by the investigating Veterinary Officer. The number of dogs in the local environment either refers to all the dogs in the compound (usually for pet dogs), or an estimate of the number of dogs in the local neighbourhood. Confirmed cases were diagnosed by the NVRI laboratory routinely using the Direct Fluorescent Antibody Test (DFAT) on tissue samples from the brain and PCR for samples from the cerebrospinal fluid. An outbreak is defined as a group of confirmed cases (one or more cases), with a clear epidemiological linkage (i.e., belonging to the same epidemiological unit such as a village, compound, or local neighbourhood).

Generally, reports of bites from suspected rabies cases are made to the local veterinary clinic, prompting a visit from the Veterinary Officer. Once a report is received, attempts are made to restrain the dog and arrive at an initial diagnosis via observation of clinical signs, such as an increase in aggressive behaviour. The criteria for sending samples to the laboratory include the following: (1) human bite from a suspected rabid dog, (2) exhibition of neurological signs similar to rabies signs, (3) research purposes where samples are collected from dog slaughterhouses where rabies tests are carried out. Most of the time, the costs associated with testing are covered by the Federal government, with a few exceptions when individuals request a test or during research projects. In some instances, dogs escape from quarantine or attempted capture and thus are not euthanised and samples could not be collected for testing. Reactive vaccination is carried out in response to an outbreak, when deemed appropriate by the local government, however, this is not implemented as policy at a national level. These reactive vaccinations are typically carried out 2 to 4 weeks after reporting. A yearly national dog vaccination campaign does occur, where vaccines are donated to owners, promoted by radio, television, flyers, and veterinarians.

**Figure 1:**
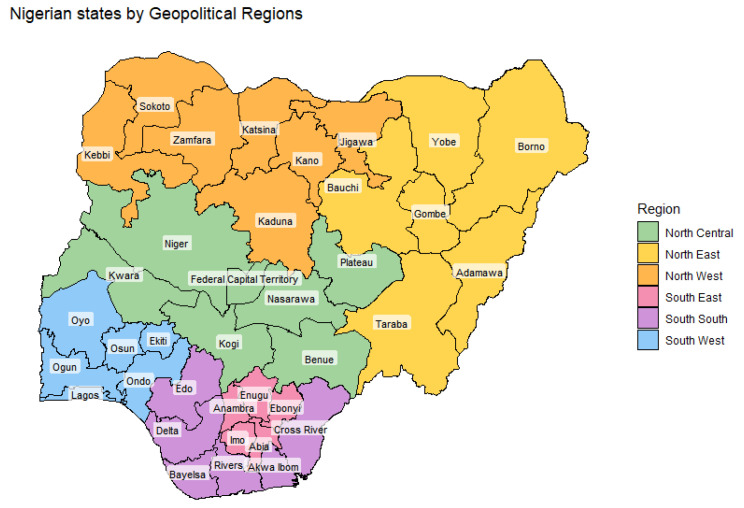
Map of Nigeria showing its states colour-coded by the six geopolitical regions.

In the analysed dataset, most outbreaks contain a single confirmed case. Data from 2014–2021 was directly sourced from FMARD, while the entries for 2022–2023 were obtained from the WAHIS database, which has a different temporal resolution. The FMARD data are aggregated at a monthly level for each individual state, containing the following information: number of reported outbreaks, susceptible animals, confirmed cases, deaths, animals killed and disposed of, and those vaccinated in response to the outbreak. The WAHIS data is aggregated twice a year (January to June and July to December) for each state. While the FMARD dataset contained exclusively dog-mediated outbreaks, the WAHIS dataset could contain outbreaks for other species (i.e., feline-mediated), but this is not reported in the dataset. Nigerian states and Geopolitical regions are shown in Figure 1.

### 2.2. Data Analysis

In the FMARD dataset, the data between 2014 and 2016 was aggregated in each state due to the small number of reports in this period. The number of susceptible dogs reported in the dataset are animals in the local environment, but as the number of dogs at the state level is unknown, it is not possible to accurately estimate rabies incidence. We present incidences assuming a 1:10 dog-to-human population ratio in the Supplementary Materials for simplicity [11,12,13]; other ratios have been discussed [14,15,16,17]. The probability of vaccination taking place after an outbreak was calculated based on the number of dog vaccination campaigns that followed the outbreak. We explored the seasonality of transmission by aggregating the number of outbreaks each month across all years (2014–2021) and conducting a SCAN test to determine the statistical significance of potential temporal clusters (threshold *p*-value = 0.05).

We developed a spatio-temporal model using NIMBLE in R for the analysis of spatial and temporal trends over the whole study period (2014–2023) combining the FMARD and WAHIS datasets. This model was adapted from the methodology developed by Moraga et al. [18]. A spatial neighbourhood matrix, W, was created to capture the connection of borders between states in Nigeria. W describes the spatial connection between states i and j, denoted by $w_{ij}$, where $w_{ij}=1$ if two states share a common border and $w_{ij}=0$ if they do not. The number of recorded outbreaks $Y_{it}$, in state i and time t, is modelled using a Poisson distribution with mean $E_{it}\theta_{it}$, where $E_{it}$ is the expected count (the average number of outbreaks across states and timepoints) and $\theta_{it}$ is the relative risk in state i and time t. We considered no reports of an outbreak in a state within a year as missing data instead of 0 outbreaks to account for the potential lack of reporting over the years.

(1) $$Y_{it}∼Poisson(E_{it}\theta_{it})$$

The logarithm of the relative risk, $\theta_{it}$, is described as the addition of the intercept, α, representing the average relative risk of the whole region, the structured spatial effect, $u_{i}$, the unstructured spatial effect, $v_{i}$, the temporal effect, $\gamma_{t}$, and the spatio-temporal random noise, $\delta_{it}$.

(2) $$log(\theta_{it})=\alpha +u_{i}+v_{i}+\gamma_{t}+\delta_{it\;}$$

We used $E_{it}$ with a uniform distribution as a prior for the intercept and a non-informative normal prior for both the unstructured spatial variation $v_{i}$ and the spatio-temporal random noise $\delta_{it}$. An improper Gaussian conditional autoregressive (CAR) distribution was used for the structured spatial variation $u_{i}$, which defines a weight modifier to each state connection based on assumption that the spatial effect of each state is dependent on effects observed in adjacent states. The temporal variation was modelled using a Random Walk Type 1 process, which implies that the value of the temporal effect $\gamma_{t}$ is based on the value at the previous time point $\gamma_{t-1}$ with a precision parameter. This assumes that changes from one time point to the next are gradual. To calculate the state-level relative risk of disease, an outbreak risk index, ${ORI}_{it}$, was calculated using the formula ${ORI}_{it}=Y_{it}/E$, with E representing the national average number of outbreaks across all states over all years, excluding states that did not report outbreaks.

R version 4.0 was used for statistical analysis and “ggplot2” [19] was used for data visualisation. The model was run in a Bayesian framework implemented in R [20] using the NIMBLE package [21], with 3 independent chains, a burn-in period of 28,000 iterations, and 20,000 samples with a thinning of 5. R scripts used for spatial modelling and SCAN analysis can be found at the following GitHub repository: https://github.com/MabEntez/Nigeria_rabies_surveillance (accessed on 24 January 2025).

## 3. Results

The FMARD data collected from 2014 to 2021 included reports of outbreaks from 29 out of 37 states (Federal Capital Territory included as an independent state), meaning that 22% of states never reported a dog-mediated rabies outbreak. There were 457 outbreaks, with 12,058 susceptible dogs in the local environment, 505 confirmed cases, 338 deaths, 155 animals killed and disposed of, and 3455 dogs vaccinated in response to outbreaks in this period (Table 1). Only in 9 out of the 457 canine outbreaks (2.0%) was there a vaccination campaign for the susceptible population carried out in response to the outbreak.

During the year with the highest number of cases reported (149), the estimated incidence was 0.79 per 100,000, Table 1. Notably, the state with the highest mean incidence of the FMARD data was Plateau state at 8.45 per 100,000 (see Supplementary Table S1). The years with the highest number of cases and outbreaks reported 0 vaccinations. A total of 3000 reported vaccinations were conducted in 2017, the year which had the second-lowest number of outbreaks, deaths, and cases. A general trend of an increase in reported outbreaks, cases, deaths, and susceptible population is noticeable over the years from 2014–2021.

The state of Plateau consistently had the highest number of reported canine rabies outbreaks throughout the period, ranging from 6 (in 2014–2016) to 110 (in 2022), Figure 2. States north of Plateau state also tend to report outbreaks consistently over the years.

To assess possible seasonality, we aggregated the number of cases and outbreaks across the years for each month (Figure 3). There was a clear increase in the number of reported outbreaks reported between July and November, with fewer reports in the months of December to June. The months of September and July showed a statistically significant increased number of reported outbreaks than expected. The months of July–September and July–November were tested as a cluster and were found to be significantly high in reported outbreaks (Supplementary Table S2).

The spatiotemporal model predicted the number of outbreaks for each state over the years, with states with very little data displaying higher variability (Supplementary Figure S5). The highest ORI across all years was found in Plateau state and its neighbouring northern states (Kaduna, Bauchi, and Kano) (Figure 4). These states were also the only states (other than Kebbi in 2017) with a calculated ORI above 1, indicating a higher outbreak risk than the expected national average (E) of 10 outbreaks per year. The temporal effect did not show any general trend over the years (Supplementary Figure S2). The model predicted the highest average temporal effect for 2022; however, all temporal effects have wide upper and lower bounds. The states with the highest mean structural variance mean were shown to be Plateau and Bauchi (Supplementary Figure S3) meaning the spatial clustering of outbreaks is found in and around these states. Unstructured variance can also be viewed (see Supplementary Figure S4), which accounts for the random noise.

## 4. Discussion

**Figure 2:**
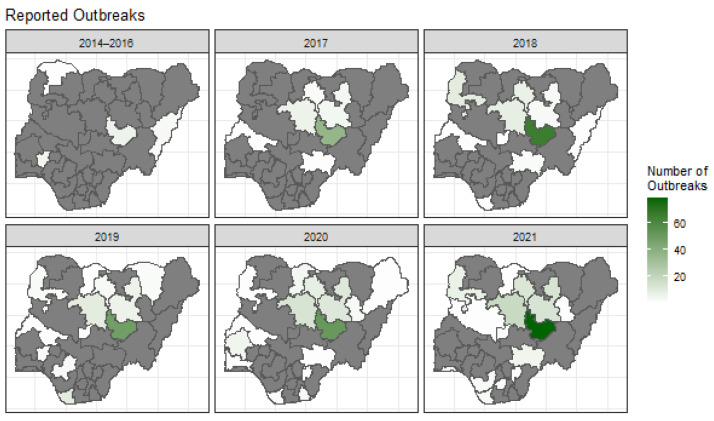
A panel of the spatial distribution of canine rabies outbreaks reported per state in Nigeria from 2014 to 2021. The data from 2014 to 2016 were combined due to the lack of reports in these years. States that did not report any outbreak are highlighted in grey.

Our state-level analysis of canine rabies outbreaks highlights changing patterns over time, with FMARD and WAHIS data underpinning our spatial models. Outbreaks were initially sparse (2014–2016) but rose markedly by 2021, illustrating the growing burden or improved reporting over this period (Figure 2). Plateau state reported substantially more outbreaks than its bordering states, which may reflect differential surveillance and the local presence of NVRI, leading to increased case submissions [22]. Previous studies support the number of outbreaks presented here, presenting a high prevalence of rabies in the north-west (50%), north-east (44%), and north-central (17%). A majority of the reported outbreaks from the FMARD data centre were around the borders of the north-west, north-east, and north-central geopolitical regions (Figure 2).

Plateau state consistently showed the highest ORI, with Bauchi, Kaduna, Kano, and Kebbi also exceeding the national average in at least one year. These findings could be an indication of the over-representation of outbreaks in specific northern states and the possibility of spatial clustering (Supplementary Figure S3). A previous study from 2012 has highlighted an increased rabies incidence in dogs in the northernmost regions of Plateau state, which borders Bauchi [23]. It has been reported using molecular epidemiological investigations that some endemic cycles occur through cross-border canine rabies transmission, potentially due to human movement with their dogs or the free movement of dogs across borders [24], indicating a need to assess the transmission of rabies between a high outbreak state and its neighbouring states. Another unknown factor in the transmission of rabies within Nigeria is the impact of wild animals, which could be another route for cross-border transmission [25,26]. The clustering of high ORI states and the heavy spatial clustering occurring could be a sign of cross-border transmission. Taking this into account brings into question the lack of outbreaks being reported in Nasarawa and Taraba and highlights the need to further investigate the disparity of outbreak reporting between states. Although fewer canine rabies studies exist in southern states, they account for more human rabies deaths, pointing to a reporting bias in the northern regions [23,27]. The general trend of increased reported outbreaks, cases, susceptible populations, and deaths is a hopeful sign of increased surveillance efforts in more recent years.

**Figure 3:**
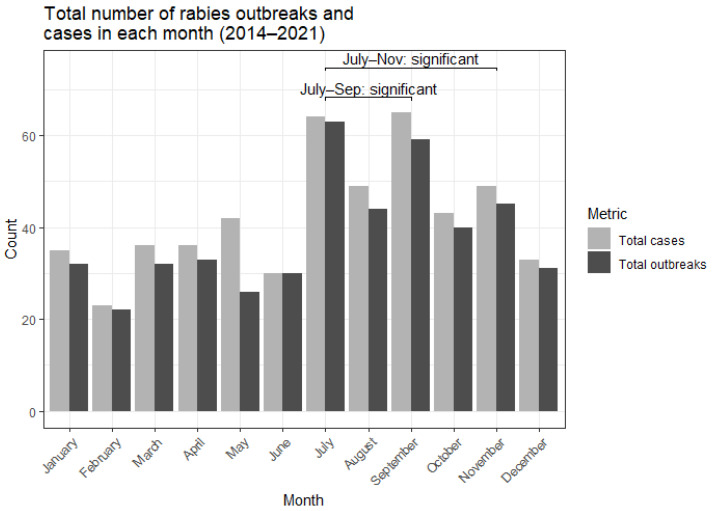
Number of canine rabies outbreaks and cases reported in Nigeria within each month from 2014 to 2021. Clusters of reported outbreaks found to be statistically significant using the SCAN test are highlighted.

**Figure 4:**
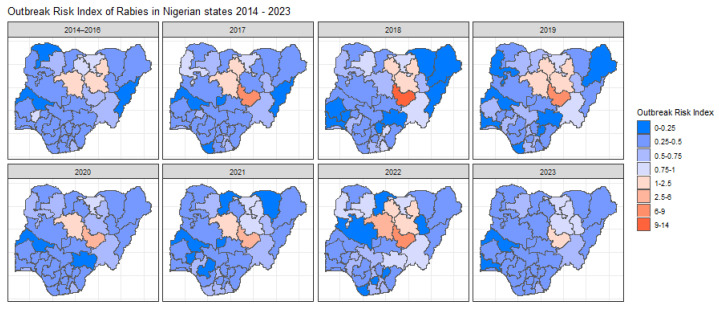
Maps illustrating the Outbreak Risk Index (ORI) for each state in Nigeria across all year ranges. These maps visualise the spatial and temporal variation in outbreak risk, highlighting regions with elevated or reduced risk based on the modelled relative risk and the observed case data. The ORI values reflect the calculated risk relative to the national average, with red shades indicating higher risk states and blue shades indicating lower risk stakes.

Compared to other times of the year, outbreaks were more frequently reported from July to September (Figure 3), corresponding to the rainy season in Nigeria. This temporal cluster of outbreaks was found to be statistically significant (Supplementary Table S2). This result aligns with previous studies based in Nigeria and Namibia that equally found an increase in reported rabies cases during the rainy season, which coincides with changes in dog ownership and dog behaviour [25,28,29]. In contrast, a retrospective study of dog bites and rabies cases in the Benue and Plateau states shows an increased number of dog bites and rabies cases in the dry season [30,31]. Due to these apparent conflicting results, further studies would need to be conducted to establish the temporal trends of rabies outbreaks in Nigeria. Collecting more granular data on factors such as age, sex, ownership status, and rural or urban environment would help clarify any seasonal patterns in dog mediated rabies transmission.

Improving centralised reporting is crucial to understanding canine rabies epidemiology across Nigeria. Currently, passive public health surveillance operates through a centralised system that inclusively engages the community. Local governments have dedicated facilities to collaborate with local communities, ensuring that reports are collected at the community level and that rural cases are captured. Nonetheless, as with all real-world applications, some gaps remain in rural settings [32]. Due to the variance of infrastructure and population density across Nigeria and evidenced by the low levels of reporting in our dataset, field-based active diagnostic surveillance [33], alongside continuous passive surveillance [34], would be required to better understand the spatial distribution of rabies in the country. The high cost of the equipment and staff training required for the current gold standard for rabies diagnosis (the fluorescent antibody test) is also preventing its uptake in developing countries [35]. Field-based rabies tests have been developed which have been shown to be sensitive, rapid diagnostic tools, whilst also being cheaper and more amenable for use in rural areas [33,36,37,38,39]. Engaging with communities has been shown to be vital, as it directly relates to increased reporting, with improved understanding and community acceptance of the value of surveillance playing an essential role [40]. In the Republic of Haiti [41], Tanzania [42,43], and Kenya [44], community-based active surveillance increased the reporting of rabies cases, identification, and resulting euthanasia of dogs that bit victims and helped identify more bite victims requiring PEP. Whilst perhaps challenging to implement nationwide, community-based surveillance is a relatively simple method of improving local surveillance records. In turn, enhancing data streams allows for more effective measures to be taken towards the future elimination of canine rabies. Furthermore, the reporting bias highlighted here is exacerbated by the centralised reporting structure of the country (Supplementary Figure S1). The NVRI is responsible for processing all rabies samples, and is located in northern Plateau state, in proximity to the states of Bauchi and Kaduna. This centralisation can introduce a reporting bias in outbreak detection. Transitioning to a decentralised system, with additional laboratories across the country, would enhance national reporting. Establishing diagnostic facilities in each geopolitical region or leveraging the existing capacity of veterinary teaching hospitals would enable more localised, rapid, and accurate reporting of rabies outbreaks.

It was calculated that only 2.0% of the outbreaks from 2014 to 2021 were followed by a dog vaccination event. The Global Alliance for Rabies Control (GARC) estimates the dog vaccination coverage in Nigeria to be 12.29% [45]. The WHO recommended target of dogs vaccinated in the susceptible population is ≥70.0% to limit or prevent the spread of a rabies outbreak [10]. Based on the GARC estimate, the dog vaccination coverage in Nigeria is insufficient to meet the WHO recommended target. Although increasing and sustaining vaccination coverage is a proactive measure that should help reduce the incidence of rabies, it has been previously reported that reactive vaccination programmes are obsolete unless good quality surveillance is already in place [46]. According to a study based in the state of Benue, despite 74% of dogs having been previously vaccinated against rabies, only 18% were up to date with their vaccinations [47]—far less than the WHO recommendation. Vaccination has been proven to be an effective method of rabies control in Bali, having achieved the recommended 70% coverage rate, however, repeated vaccination campaigns would still be necessary to eliminate canine rabies in Bali [48]. Mass vaccination campaigns, in addition to the surveillance recommendations mentioned above, will bolster rabies control by reducing transmission, enabling earlier outbreak detection, and facilitating timely, targeted public health interventions.

Another challenge in Nigeria is understanding the dog population at risk, both for estimating vaccination campaign targets and for estimating the incidence of the disease. The only information currently available regarding the susceptible dog population is the best estimate of dogs in the local environment, as reported by the Veterinary Officer. However, since the data analysis conducted in this paper, dog population numbers have become available [49]. Historically, a 1:10 dog-to-human ratio has been used to approximate dog populations in a country [11,12], Supplementary Table S1. However, other approaches, such as the creation of a ‘dog density’ map in Tanzania, have helped to effectively direct vaccination campaigns [50]. This was achieved by identifying factors of dog ownership (such as socio-economic status and size of household) to infer the dog population. Such an activity is relatively cheap to carry out and would enhance the understanding of rabies spread in the country.

A One Health approach has been seen to help control rabies and would simultaneously help improve understanding of rabies epidemiology. A study based in Plateau state argued that creating a One Health initiative would ensure sustainable rabies control state-wide, through improved awareness of the disease and uptake of dog vaccinations [51]. Following an integrated bite-case management system would help improve information sharing between human and veterinary services and identification of bite-exposure victims requiring PEP as well as the identification of the suspected rabid dog [52].

There were several limitations whilst analysing this dataset. Primarily, it was acknowledged that reporting at a local government level has been very challenging, impacting how states are reporting. It is worth noting that the lack of cases in states bordering those with many cases further suggests it is at least in part a reporting problem. Another limitation is that the data are already aggregated for each month, therefore the exact number of cases in each outbreak is unknown in months where more than one outbreak is reported. This also makes it difficult to determine if a particular intervention (such as dog vaccination) is tied to a certain outbreak from a particular month if there is more than one outbreak reported within that month. The records do not differentiate between ‘suspected’ and ’confirmed’ cases, which impacts how we interpret them. It was also not possible to identify transmission events in the sylvatic population, where rabies is endemic. The ’susceptible population’ is based on the reporting Veterinarian Officer’s estimate of the local population, which is likely underestimated in Nigeria, where a substantial number of dogs are free-roaming [23,53]. Frequent omissions of this estimate in our dataset hindered the accurate calculation of vaccination coverage during outbreaks. Improved estimation of the susceptible population is essential for enhancing rabies surveillance.

## 5. Conclusions

In this study, we summarised a decade (2014–2021) of dog-mediated rabies outbreaks and assessed their spatial and temporal trends. Our analysis revealed a higher incidence of reported outbreaks in the North Central region, with Plateau state and Bauchi states identified as high-risk areas through spatiotemporal modelling—suggesting potential under-reporting in neighbouring regions. We also observed an increase in outbreaks during the rainy season (July to September). These findings highlight the urgent need to intensify and update the current surveillance system. We recommend decentralising diagnostic services by expanding capacity beyond the single reference laboratory, utilising additional laboratories and the existing infrastructure at veterinary teaching hospitals. Integrating rapid field test kits and community-based active surveillance with the current passive system will further enhance early outbreak detection and reporting. In addition, mass vaccination campaigns to reach the >70% WHO recommended target, implemented alongside these enhanced surveillance measures, will bolster rabies control by reducing transmission and facilitating timely, targeted public health interventions.

## Figures and Tables

**Table 1:** Yearly summary across all states of the FMARD data from 2014–2021.

Year	Total Outbreaks	Total Cases	Total Deaths	Total Susceptible	Total Killed Disposed	Total Vaccinated
		(Estimated Incidence)				
2014–2016	9	15 (0.08)	11	32	4	165
2017	50	50 (0.27)	23	683	27	3000
2018	84	98 (0.52)	83	579	10	80
2019	73	87 (0.46)	73	433	9	210
2020	99	106 (0.56)	71	3519	31	0
2021	142	149 (0.79)	76	6812	74	0
Total	457	505	337	12058	155	3455

## Data Availability

The R scripts presented in the study are openly available in https://github.com/MabEntez/Nigeria_rabies_surveillance (accessed on 28 February 2025). Data were also derived from the following resources available in the public domain: wahis.woah.org (accessed on 28 February 2025). Further data supporting the conclusions of this article will be made available by the authors on request.

## Supplementary Materials

The following supporting information can be downloaded at: https://www.mdpi.com/article/10.3390/tropicalmed10030076/s1, Table S1: Number of confirmed cases per 100,000 dogs in each state in Nigeria from 2014 to 2021; Table S2: Results from the SCAN Statistic to determine the significance of the temporal clusters identified in Figure 2; Figure S1: Flowchart displaying the channel of animal disease reporting in Nigeria; Figure S2: Average temporal effect predicted by the spatiotemporal model (2014–2021); Figure S3: Average structured spatial effect predicted by the spatiotemporal model (2014–2021); Figure S4: Average unstructured spatial effect predicted by the spatiotemporal model (2014–2021). Figure S5: The mean and 95% quantiles of predicted outbreak counts across Nigerian states (2014–2023).

## Abbreviations

The following abbreviations are used in this manuscript:

NVRI	National Veterinary Research Institute
CVON	Chief Veterinary Officer of Nigeria
DVS	Director of Veterinary Services
PEP	Post-exposure prophylaxis
FMARD	Federal Ministry of Agriculture and Rural Development
FAT	Fluorescent Antibody Test
CAR	Gaussian conditional autoregressive
FCT	Federal Capital Territory
ORI	Outbreak Relative Index
GARC	Global Alliance for Rabies Control
