# Supplementary material for: Dog-Mediated Rabies Surveillance in Nigeria (2014–2023): Investigating Seasonality and Spatial Clustering
Source: Trop Med Infect Dis. 2025 Mar 12;10(3):76. doi: 10.3390/tropicalmed10030076 (PMC11946520; doi:10.3390/tropicalmed10030076)
Supplement: Supplementary file 1 [file tropicalmed-10-00076-s001.zip › tropicalmed-3471188-supplementary.pdf]

Supplementary Materials

Table S1: Number of confirmed cases per 100,000 dogs in each state in Nigeria from 2014 to 2021. Population density was estimated using a 1:10 dog-to-human ratio, based on 2016 human population data. The yearly number of confirmed rabies cases (per 100,000 dogs) is shown for each state, along with the mean incidence over the 2014–2021 period.

State	Estimated Population	2014	2015	2016	2017	2018	2019	2020	2021	Mean Cases 2014 - 2021
Abia	372,734.7	0 (0)	0 (0)	0 (0)	0 (0)	0 (0)	0 (0)	0 (0)	0 (0)	0 (0)
Adamawa	424,843.6	0 (0)	3 (0.71)	0 (0)	0 (0)	1 (0.24)	0 (0)	0 (0)	0 (0)	0.5 (0.12)
Akwa Ibom	548,217.7	0 (0)	0 (0)	0 (0)	0 (0)	0 (0)	0 (0)	3 (0.55)	0 (0)	0.375 (0.07)
Anambra	552,780.9	0 (0)	0 (0)	0 (0)	0 (0)	0 (0)	0 (0)	0 (0)	0 (0)	0 (0)
Bauchi	653,731.4	0 (0)	0 (0)	0 (0)	4 (0.61)	3 (0.46)	6 (0.92)	11 (1.68)	13 (1.99)	4.625 (0.71)
Bayelsa	227,796.1	0 (0)	0 (0)	0 (0)	0 (0)	1 (0.44)	9 (3.95)	2 (0.88)	3 (1.32)	1.875 (0.82)
Borno	586,018.3	0 (0)	0 (0)	0 (0)	0 (0)	0 (0)	0 (0)	1 (0.17)	0 (0)	0.125 (0.02)
Cross River	386,626.9	0 (0)	0 (0)	0 (0)	0 (0)	0 (0)	0 (0)	0 (0)	0 (0)	0 (0)
Delta	566,336.2	0 (0)	0 (0)	0 (0)	0 (0)	0 (0)	0 (0)	0 (0)	3 (0.53)	0.375 (0.07)
Ebonyi	288,038.3	0 (0)	0 (0)	0 (0)	0 (0)	0 (0)	0 (0)	1 (0.35)	1 (0.35)	0.25 (0.09)
Edo	423,559.5	0 (0)	0 (0)	0 (0)	0 (0)	0 (0)	3 (0.71)	0 (0)	0 (0)	0.375 (0.09)
Ekiti	327,079.8	0 (0)	0 (0)	0 (0)	0 (0)	0 (0)	0 (0)	0 (0)	0 (0)	0 (0)
Enuga	441,111.9	0 (0)	0 (0)	0 (0)	0 (0)	0 (0)	0 (0)	0 (0)	0 (0)	0 (0)
FCT	356,412.6	0 (0)	0 (0)	0 (0)	0 (0)	1 (0.28)	1 (0.28)	0 (0)	2 (0.56)	0.5 (0.14)
Gombe	325,696.2	0 (0)	0 (0)	0 (0)	0 (0)	0 (0)	0 (0)	3 (0.92)	1 (0.31)	0.5 (0.15)
Imo	540,875.6	0 (0)	0 (0)	0 (0)	0 (0)	0 (0)	0 (0)	0 (0)	1 (0.18)	0.125 (0.02)
Jigawa	582,816.3	0 (0)	0 (0)	0 (0)	0 (0)	0 (0)	0 (0)	0 (0)	0 (0)	0 (0)
Kaduna	825,236.6	0 (0)	0 (0)	0 (0)	6 (0.73)	8 (0.97)	9 (1.09)	13 (1.58)	18 (2.18)	6.75 (0.82)
Kano	1,307,689	0 (0)	0 (0)	0 (0)	2 (0.15)	7 (0.54)	3 (0.23)	8 (0.61)	12 (0.92)	4 (0.31)
Katsina	783,131.9	0 (0)	0 (0)	0 (0)	0 (0)	0 (0)	1 (0.13)	4 (0.51)	1 (0.13)	0.75 (0.1)
Kebbi	444,005	0 (0)	0 (0)	0 (0)	0 (0)	9 (2.03)	2 (0.45)	1 (0.23)	7 (1.58)	2.375 (0.53)
Kogi	447,349	0 (0)	0 (0)	0 (0)	0 (0)	0 (0)	0 (0)	0 (0)	0 (0)	0 (0)
Kwara	319,289.3	0 (0)	0 (0)	0 (0)	1 (0.31)	1 (0.31)	1 (0.31)	1 (0.31)	0 (0)	0.5 (0.16)
Lagos	1,255,060	0 (0)	0 (0)	0 (0)	0 (0)	0 (0)	0 (0)	0 (0)	3 (0.24)	0.375 (0.03)
Nasarawa	252,339.5	0 (0)	0 (0)	0 (0)	0 (0)	0 (0)	0 (0)	0 (0)	0 (0)	0 (0)
Niger	555,624.7	0 (0)	0 (0)	0 (0)	0 (0)	0 (0)	0 (0)	0 (0)	1 (0.18)	0.125 (0.02)
Ogun	521,771.6	0 (0)	0 (0)	0 (0)	0 (0)	0 (0)	0 (0)	1 (0.19)	0 (0)	0.125 (0.02)
Ondo	467,169.5	0 (0)	0 (0)	0 (0)	0 (0)	0 (0)	0 (0)	0 (0)	0 (0)	0 (0)
Osun	470,558.9	3 (0.64)	2 (0.43)	0 (0)	0 (0)	0 (0)	2 (0.43)	0 (0)	0 (0)	0.875 (0.19)
Oyo	784,086.4	0 (0)	0 (0)	0 (0)	0 (0)	0 (0)	0 (0)	5 (0.64)	0 (0)	0.625 (0.08)
Plateau	420,044.2	6 (1.43)	0 (0)	0 (0)	36 (8.57)	65 (15.47)	48 (11.43)	51 (12.14)	78 (18.57)	35.5 (8.45)
Rivers	730,392.4	0 (0)	0 (0)	0 (0)	0 (0)	0 (0)	0 (0)	0 (0)	0 (0)	0 (0)
Sokoto	499,809	0 (0)	0 (0)	1 (0.2)	0 (0)	0 (0)	0 (0)	0 (0)	0 (0)	0.125 (0.03)
Taraba	306,683.4	0 (0)	0 (0)	0 (0)	0 (0)	0 (0)	0 (0)	0 (0)	0 (0)	0 (0)
Yobe	329,413.7	0 (0)	0 (0)	0 (0)	0 (0)	0 (0)	2 (0.61)	0 (0)	0 (0)	0.25 (0.08)
Zamfara	451,542.7	0 (0)	0 (0)	0 (0)	0 (0)	0 (0)	0 (0)	0 (0)	0 (0)	0 (0)

Table S2: Results from the SCAN Statistic to determine the significance of the temporal clusters identified in Figure 2. Monthly outbreak data (2014–2021) were tested for evidence of temporal clustering using a SCAN Statistic. This table shows the months or sequences of months tested, the number of outbreaks in each cluster, and the corresponding p-values, indicating whether the cluster is statistically significant ($p < 0.05$).

Cluster	Months	Outbreaks	Total Outbreaks	Probability	P value
T1	July	84	638	0.00001	3.96E-05*
T2	September	80		0.00007	3.18E-04*
T3	October	64		0.01685	1.32E-01
T4	November	64		0.01685	1.32E-01
T5	July - September	224		0	1.25E-08*
T6	July - November	352		0	8.64E-12*

FLOW OF ANIMAL DISEASE REPORTING IN NIGERIA

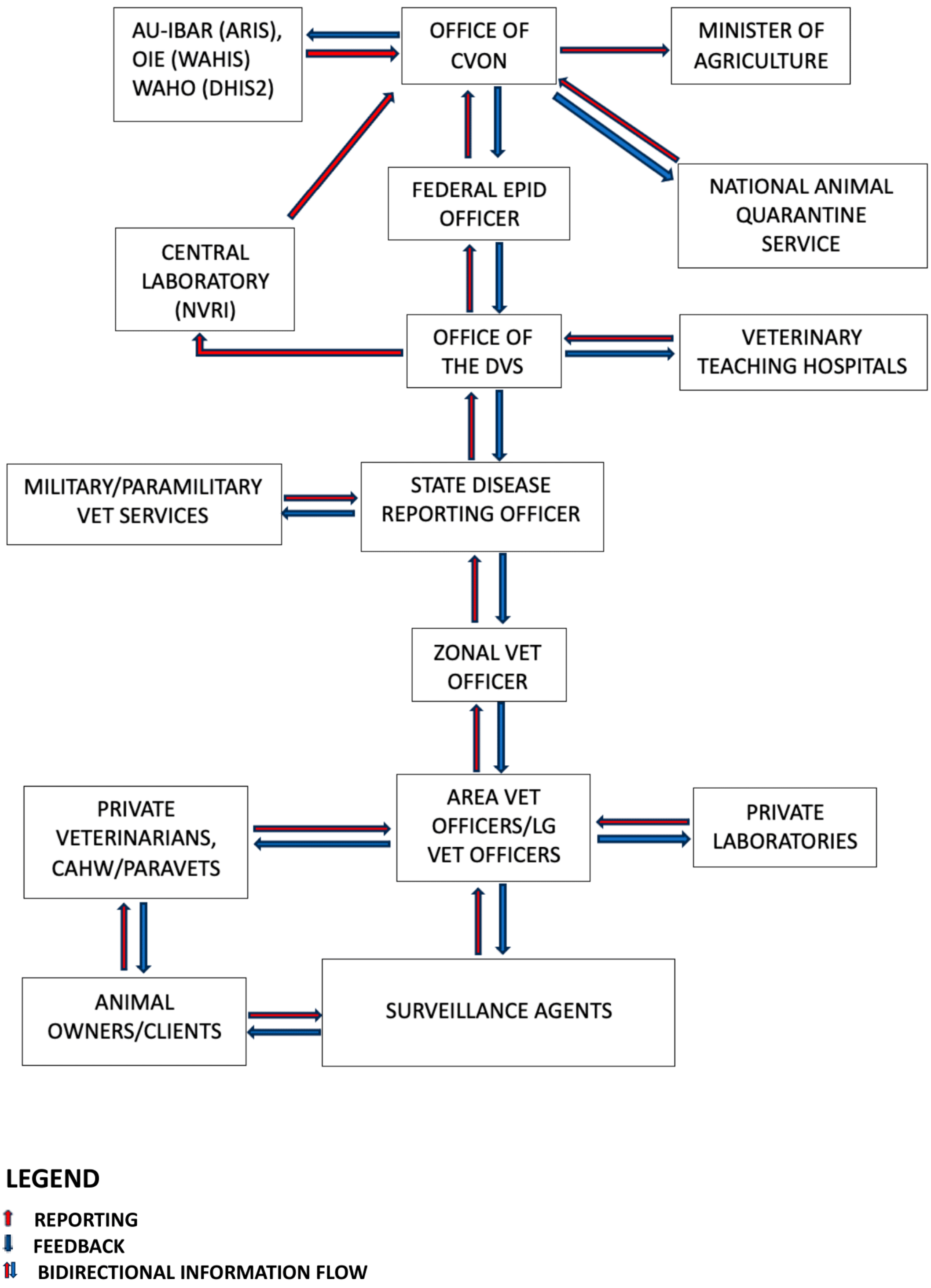

Figure S1: Flowchart displaying the channel of animal disease reporting in Nigeria. A schematic illustration of the passive surveillance process for animal rabies. It shows how cases are notified to the central authorities—

from the local community and state Directors of Veterinary Services (DVS) to the office of the Chief Veterinary Officer of Nigeria (CVON)—and how this information is then cascaded back to local communities. Acronym Defenitions are explain below:

AU-IBAR – African Union – Inter-African Bureau for Animal Resources

ARIS – Animal Resources Information System

OIE – World Organisation for Animal Health

WAHIS – World Animal Health Information System

WAHO – West African Health Organisation

DHIS$_2$ – District Health Information Software 2

CAHW – Community Animal Health Workers

PARAVET – Paraprofessional Veterinarians

NVRI – National Veterinary Research Institute

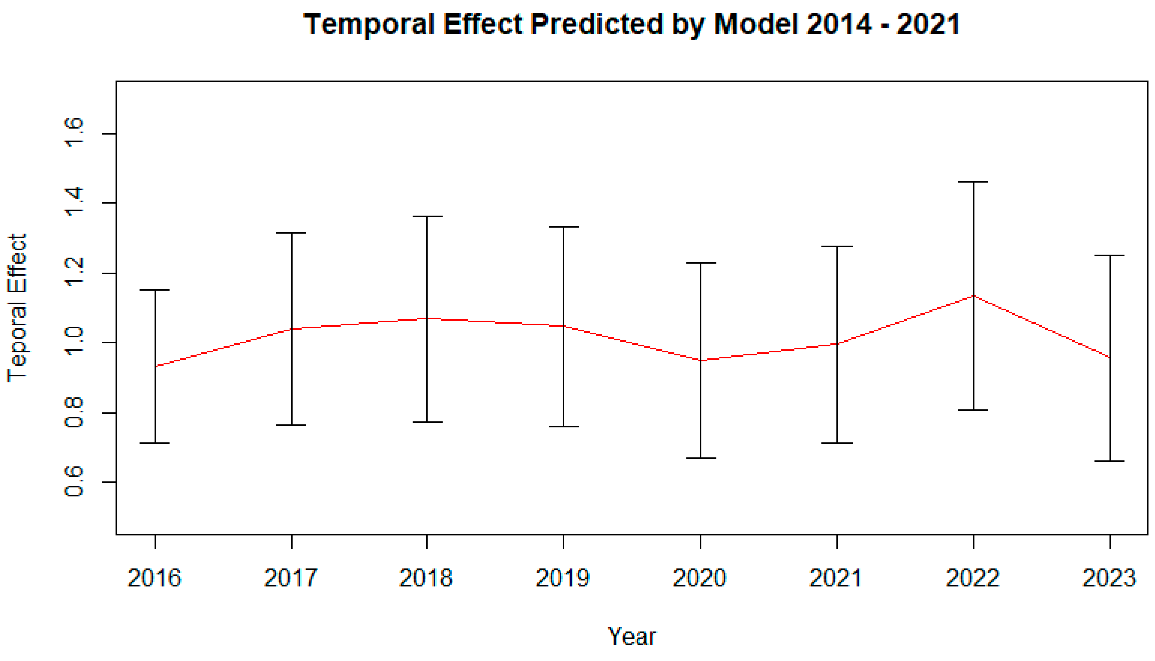

Figure S2: Average temporal effect predicted by the spatiotemporal model (2014–2021). This figure shows the modelled temporal effect for canine rabies outbreaks across all Nigerian states, highlighting year-to-year variations. The average effect for each year indicates whether the likelihood of outbreaks was higher, lower, or about average compared to other years in the study period, with 95% confidence intervals.

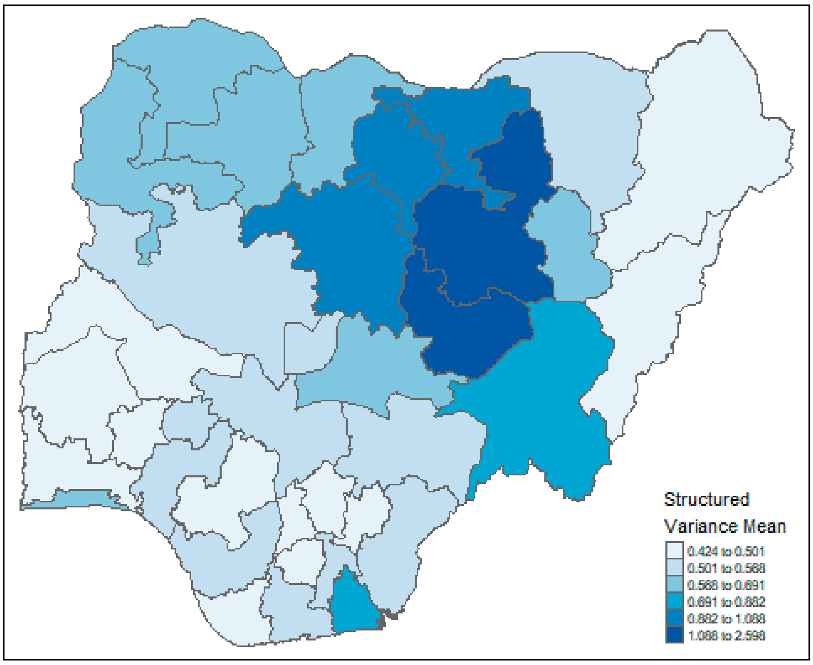

Figure S3: Average structured spatial effect predicted by the spatiotemporal model (2014–2021). A map of Nigeria illustrating the structured spatial effect for each state. States in dark blue have higher spatial effect values, indicating a comparatively elevated baseline risk of outbreaks (after accounting for other factors). This structured effect captures how neighbouring states' outbreak patterns influence each other.

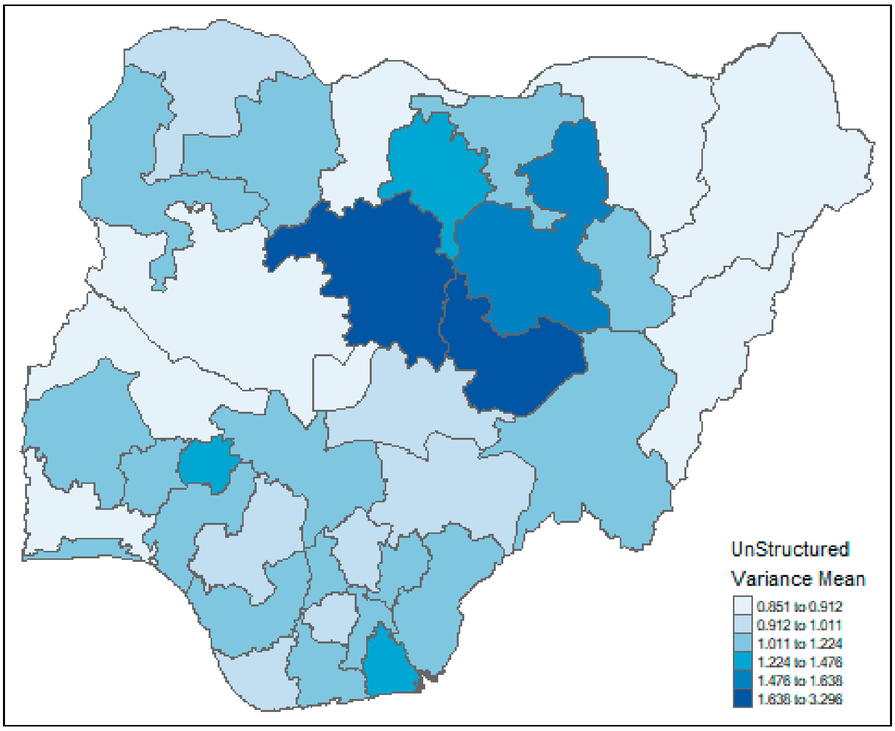

Figure S4: Average unstructured spatial effect predicted by the spatiotemporal model (2014–2021). A map representing random (unstructured) spatial variation in canine rabies outbreaks after controlling for the baseline and structured effects. This figure highlights any additional location-specific risk that cannot be explained by neighbourhood relationships or broader spatial patterns.

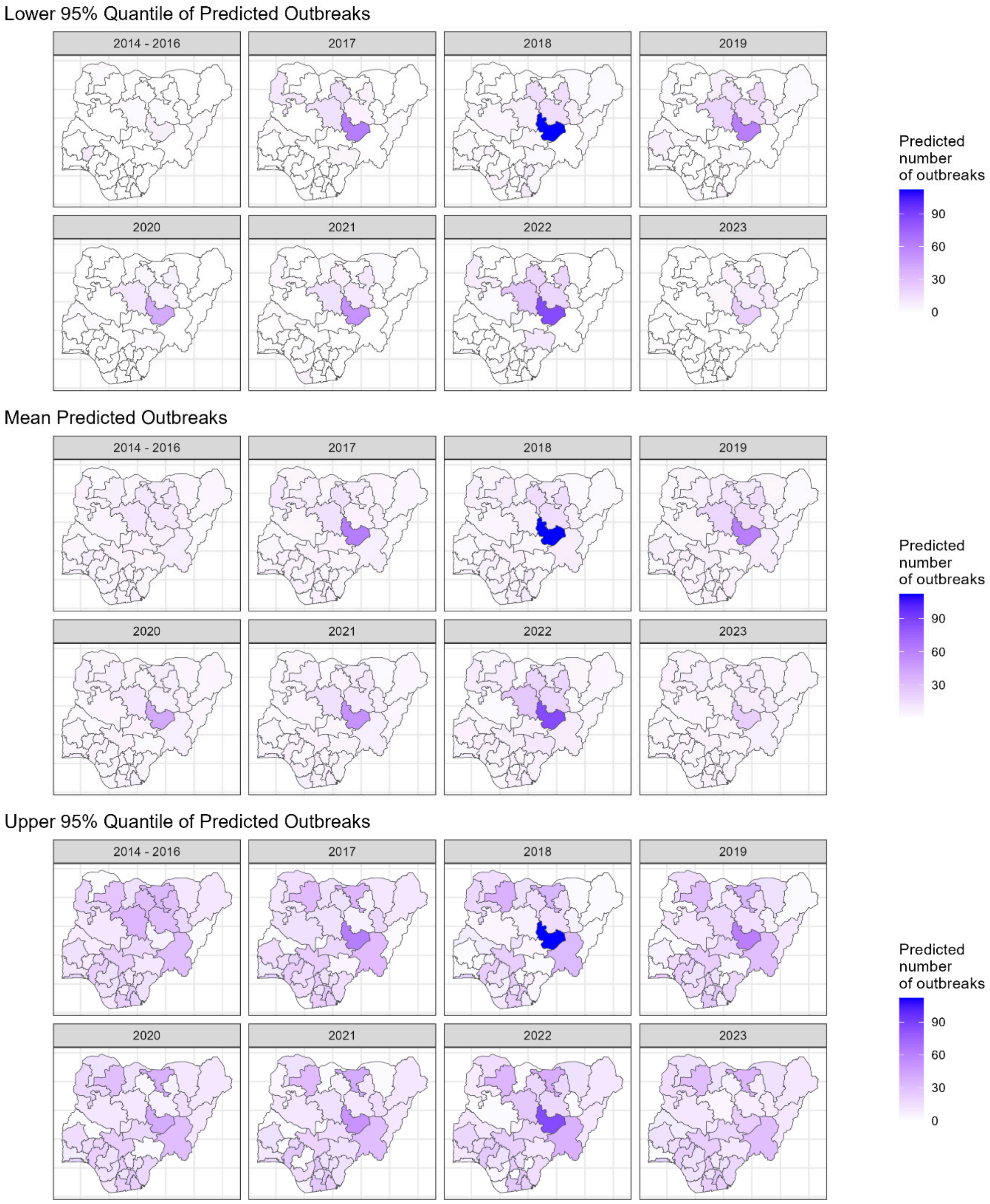

Figure S5: The mean and 95% quantiles of predicted outbreak counts across Nigerian states (2014–2023). Maps showing the lower and upper 95% quantiles of predicted canine rabies outbreaks from the spatiotemporal model. These estimates encompass uncertainty in the model's predictions, illustrating the potential range of outbreak counts for each state over the study period.
